# High-Speed and Direction-Controlled Formation of Silicon Nanowire Arrays Assisted by Electric Field

**DOI:** 10.1186/s11671-020-3259-5

**Published:** 2020-01-30

**Authors:** Pin-Ju Chien, Ta-Cheng Wei, Chia-Yun Chen

**Affiliations:** 1 Department of Materials Science and Engineering, National Cheng Kung University, Tainan, 70101 Taiwan; 2 Hierarchical Green-Energy Materials (Hi-GEM) Research Center, National Cheng Kung University, Tainan, 70101 Taiwan

**Keywords:** Etching of Si, Formation mechanism, Reflectivity, Surface wettability

## Abstract

Metal-assisted chemical etching (MaCE), a low-cost and versatile method was considered a promising technique for preparing silicon nanowires (SiNWs), yet the lack of well controlling the injected holes within Si might reduce the etching rate, create the unwanted sidewall etching, and degrade the structural uniformity. Herein, in this study, the bias-modulated MaCE process was performed, showing the etching rates more than four times of magnitude than that of typical bias-free MaCE with large-area uniformity. It was found that the field-mediated hole rectification overwhelmed the effect of retarded diffusivity from reactive ions, and thus the dynamics of distributed etching were therefore transferred to the directional etching behaviors. In addition, the etching orientation could be also manipulated with the external bias. The results demonstrated that the etching direction was switched toward the slanted features by varying the electric polarization, creating the special slanted/vertical NW arrays, which possessed the superior antireflection characteristics than the conventional vertically aligned features.

## Introduction

Low-dimensional silicon (Si) nanostructures displayed remarkable electronic, mechanical, and optoelectronic properties that could act as building elements of functional devices and applications [1–3], such as field-effect transistors, biosensors, and photovoltaic cells [4–9]. To form the regular arrays of Si nanostructures, metal-assisted chemical etching has been considered the prevailing strategy that even enabled to form Si nanowire (SiNW) arrays on planar substrates [10, 11], powders [12, 13], and pyramidal structures [14]. In the etching process, the hole injections across metal catalysts toward Si underneath and subsequently the dissolution of oxidized Si were continuously occurred, thus resulting in the long etching pores. However, the generated holes could diffuse within Si matrix rather than always moved vertically with respect to the substrate plane; it usually caused the formation of numerous Si nanopores near the primary etching sites, leaving the uncontrollable etching profiles behind [15]. This feature turned out to be particularly obvious while the solutions owned high viscosity.

The lack of well controlling the injected holes within Si might hinder the practical applications of Si nanostructures for practical use.

To overcome this demanding issue, the employment of the external field seemed to be promising. Liyi Li et al., demonstrated that the high aspect ratio (> 10:1) regular hole or strip arrays could be realized by using electric bias-attenuated MaCE, but the feature dimensions were in microscale [16]. Inspired by this work, in this study, we attempted to explore the feasibility of fabricating SiNW arrays by means of applying bias during MaCE process. Both positive and negative bias were investigated to understand the field-dependent etching kinetics. In addition to the etching rate, we found that the etching orientation was able to be modulated, where the dual-segment SiNWs in the form of slanted/vertical features were realized by tuning the bias direction. The underlying etching mechanism and etching kinetics in the presence of electric field were explored, and the superior antireflection characteristics of unique slanted/vertical SiNW arrays were presented in both experimental and simulated analysis.

## Methods

### Substrate Preparation

The single crystalline (100), single-side polished Si substrates with a thickness of 525 μm were used as starting materials. The Si substrates were ultrasonically cleaned in isopropyl alcohol, acetone, and deionized water for several cycles, and then were further cleaned in SC-1 solution (1 part of NH_4_OH, 1 part of H_2_O_2_, and 5 part of deionized water) for 30 min to clean the surfaces and resulted in the hydrophilic surfaces.

### Nanosphere Lithography

In order to fabricate the highly ordered aligned nanostructures, nanosphere lithography (NSL) was conducted. Basically, polystyrene nanospheres (PSs) with diameters of 300 nm are slowly dispersed and assembled in the hexagonal close-packed features at the air/water interfaces in the Petri dish, and then directly transferred onto the Si substrates. The size shrinkage of large-scale and uniform PSs was achieved by employing oxygen plasma with a power of 100 W under a process pressure of 200 mTorr. The etching time was set to be 120 s and the oxygen flow was maintained at 12 sccm. Subsequently, 30 nm silver film was deposited using an electron beam evaporator at a rate of 0.3 Å/s under the vacuum condition of 7.0 × 10^−6^ Torr. Afterward, the remaining PSs were completely removed by sonication for 2 h in the toluene, which resulted in the formation of patterned silver mesh on Si substrates.

### Fabrication of Si Nanowires

The as-cleaned Si substrates were pasted with copper tapes as the electrodes as top and rear side and connected with power supply for applying the electric field. The applied voltages were adjusted within 40 V to 40 V. The Si substrates with the loadings of either Ag nanoparticles or patterned Ag layers were formed when immersing them in an etching mixture consisting of HF (49%), H_2_O_2_ (30%), and DI water with the concentrations of 4 M and 0.28 M, respectively [17–20]. After conducting the etching process, the residual Ag layers were removed with the concentrated HNO_3_ (65%)

### Characterizations

Morphologies of as-formed nanowires were characterized by field emission scanning electron microscopy (SEM, LEO 1530). Contact angle analysis was obtained using a Theta Lite (TL101). Luminescent behaviors of SiNWs were characterized through a photoluminescent (PL) system equipped with a light-emitting diode lamp (output power: 780 mW) and the wavelength of the light source was 365 nm. UV/Vis reflectance spectra were recorded by a UV-vis-NIR spectrophotometer (Varian, Cary 5000, Australia). The optical reflectance was simulated with finite-difference time-domain (FDTD), where the perfectly matched boundary was selected along the illumination directions.

## Results and Discussion

**Fig. 1:**
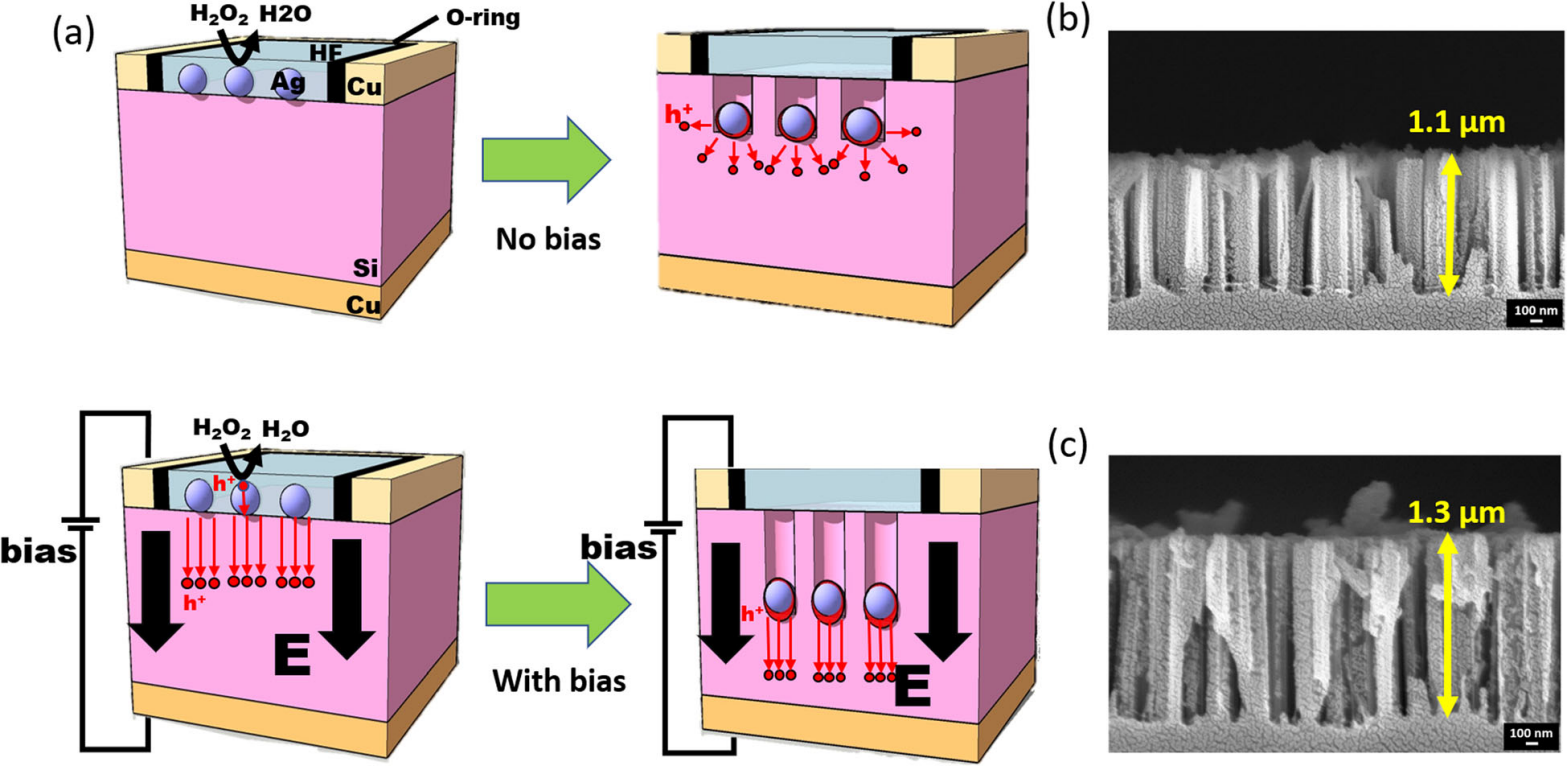
**a** Schematic illustrations presenting the typical MaCE (figure above) and bias-assisted MaCE (figure below). Cross-sectional SEM images of SiNWs made by **b** typical MaCE and **c** bias-assisted MaCE (+ 10 V)

Comparisons of typical MaCE and bias-assisted MaCE employed on the Ag-loaded Si substrates were presented in Fig. 1a. It could be found that the H_2_O_2_ oxidants provided the holes diffusing toward Si assisted by the existed Ag catalysts. The injection of holes, nevertheless, was not always forwarded in unidirectional orientation, which instead were spread out owing from the fact that the uneven facets of AgNPs in contact with Si, as shown in Fig. 1a. These features explicitly caused the loss of holes that were incapable of contributing to the directional dissolution of Si for NW formation but instead being distributed and might readily lead to the formation of porous structures. By contrast, the employment of forward bias (+ 10 V) in vertical arrangement with respect to the substrate planes could dramatically modify the etching kinetics. The correlated design of field-applied MaCE was schematically presented in Additional file 1: Figure S1. In fact, the involved polarization potential enforced all the holes diffusing along the orientation of applied field that responded to the enhanced etching rates (260 nm/min) (Fig. 1c) compared with typical MaCE process (220 nm/min) (Fig. 1b). The variation of positive bias applied within MaCE process could therefore resulted in the change of etching rates, as demonstrated in Additional file 1: Figure S2, where it clearly evidenced the hole rectification of MaCE as the positive bias was involved.

**Fig. 2:**
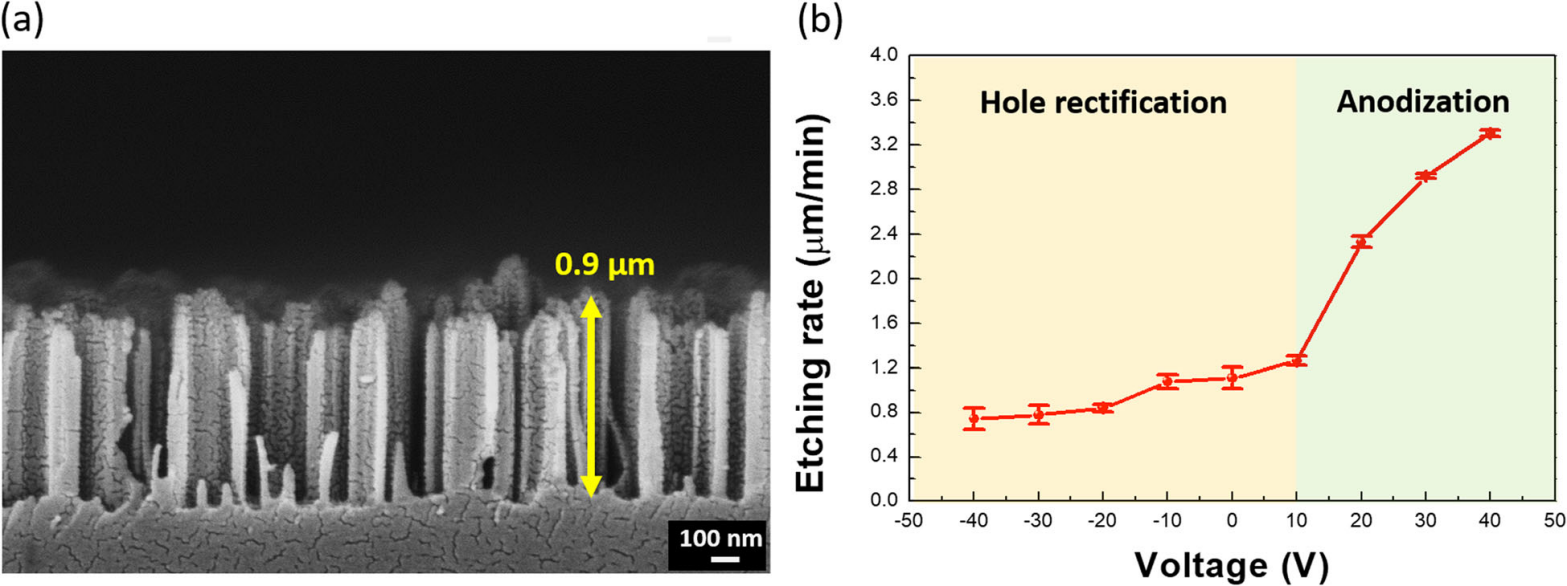
**a** Cross-sectional SEM image of SiNWs obtained from bias-assisted MaCE with − 10 V of applied electric field. **b** Relationship of applied voltage and corresponded etching rate for the formation of SiNWs

Such field-assisted effect, on the other hand, was also valid while the MaCE process was subjected to the negative bias. Figure 2a indicated the opposite rectification of hole diffusion with respect to the hole injection path, which inhibited the effective dissolution of Si by constraining the holes within Ag microscopic electrodes and gave rise to the reduced etching rate (180 nm/min). In addition, the overall range of applied bias on the etching rate of MaCE reactions was demonstrated in Fig. 2b. It indicated the transition of etching kinetics with respect to the polarization of involved bias. The negative bias unambiguously caused the reduction of etching rate, whereas the positive bias at + 10 V facilitated the effective etching of Si directionally through hole rectification effect and reflected the gradual increase of etching rate. With larger bias, in addition to the hole rectification, it potentially introduced the newly generated holes involving in the directional etching of Si, which was correlated with anodization effect [21, 22]. Such effect predominately controlled the etching kinetics, thus dramatically enhanced the etching rates more than four times of magnitude than that of typical bias-free MaCE. We therefore could conclude that the combined effects of hole rectification and anodization took place on Si that could modulate the etching kinetics and correlated behaviors.

**Fig. 3:**
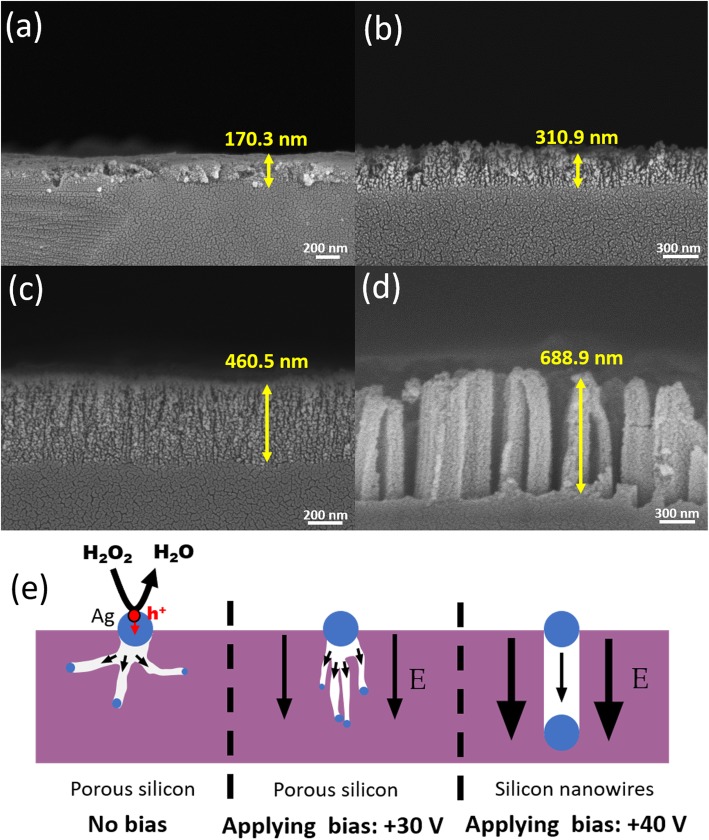
Cross-sectional SEM images of Si nanostructures obtained from **a** MacE without bias, **b** MaCE with + 20 V, **c** MaCE with + 30 V, and **d** MaCE with + 40 V. **e** Schematic illustrations of nanostructure formation under various bias conditions

To further unveil the bias-modulated kinetics in MaCE reaction, the comparably high viscous solutions were utilized. This was accomplished by mixing etching regents in 90% of IPA solvents. The relationship between the diffusion coefficient and the viscosity could be expressed as below [23],

1 $$ \mathrm{D}=\mathrm{AT}/{\upeta}^{\mathrm{p}} $$

in which *D* is the diffusion coefficient, *A* is the empirical constant, *T* the temperature, η is the viscosity of solvent, and p the viscosity exponent. Accordingly, the viscosity of IPA solvent is 2.1 mPa s at 25 ^°^C, which is more than 2.3 times larger than that of water (viscosity = 0.9 mPa s). Therefore, one can expect the ion diffusivities of both H_2_O_2_ and F− ions in IPA medium were much lower than those in water condition. As indicated in Fig. 3a, the thin porous structures with a thickness of 170.3 nm were created under a 5-min etching when no bias was employed. This was contributed by the fact that the involved IPA solvents possessing a large viscosity that intended to spread out the holes in a random orientation, and thus the porous features rather than one-dimensional structures were formed. To facilitate the charge accumulation for initiating vertical etching of Si, the various positive biases were introduced, as shown in Fig. 3b–d. It should be noted that at low involved bias including + 20 V and + 30 V, the film thickness of porous structures was clearly increased, leading to the enhanced etching rates from 34.0 nm/min (0 V), 62.2 nm/min (+ 20 V) toward 92.1 nm/min (+ 30 V).

These results validated the effect of bias that essentially dominates the etching kinetics, where most of the separated holes were assumed to be collected and accumulated just beneath sites of Ag catalysts, thus initiating the deeper etching morphologies. When the applied bias was increased to + 40 V, the field-mediated hole rectification turned out to overwhelm the influences on retarded diffusivity of reactive ions, the dynamics of random and distributed etching were therefore transferred to the directional etching behaviors; the vertically aligned SiNW arrays were realized, showing the highest etching rates up to 137.8 nm/min among these four testes conditions, as presented in Fig. 3d. The distinct formation mechanism with respect to the applied bias could be understood from Fig. 3e. It indicated that the isotropic diffusion pathways of hole led to the formation of the thin porous film when no bias was introduced. By contrast, the relatively anisotropic hole transport was found under the involvement of modest bias, where the generated pores eventually moved along the bias orientation and established the features of multiple pores. At high bias condition, the injected holes were enforced to accumulate at catalyst/Si interfaces and synergistically move into Si following the polarization field, thus creating the vertically etched profiles.

**Fig. 4:**
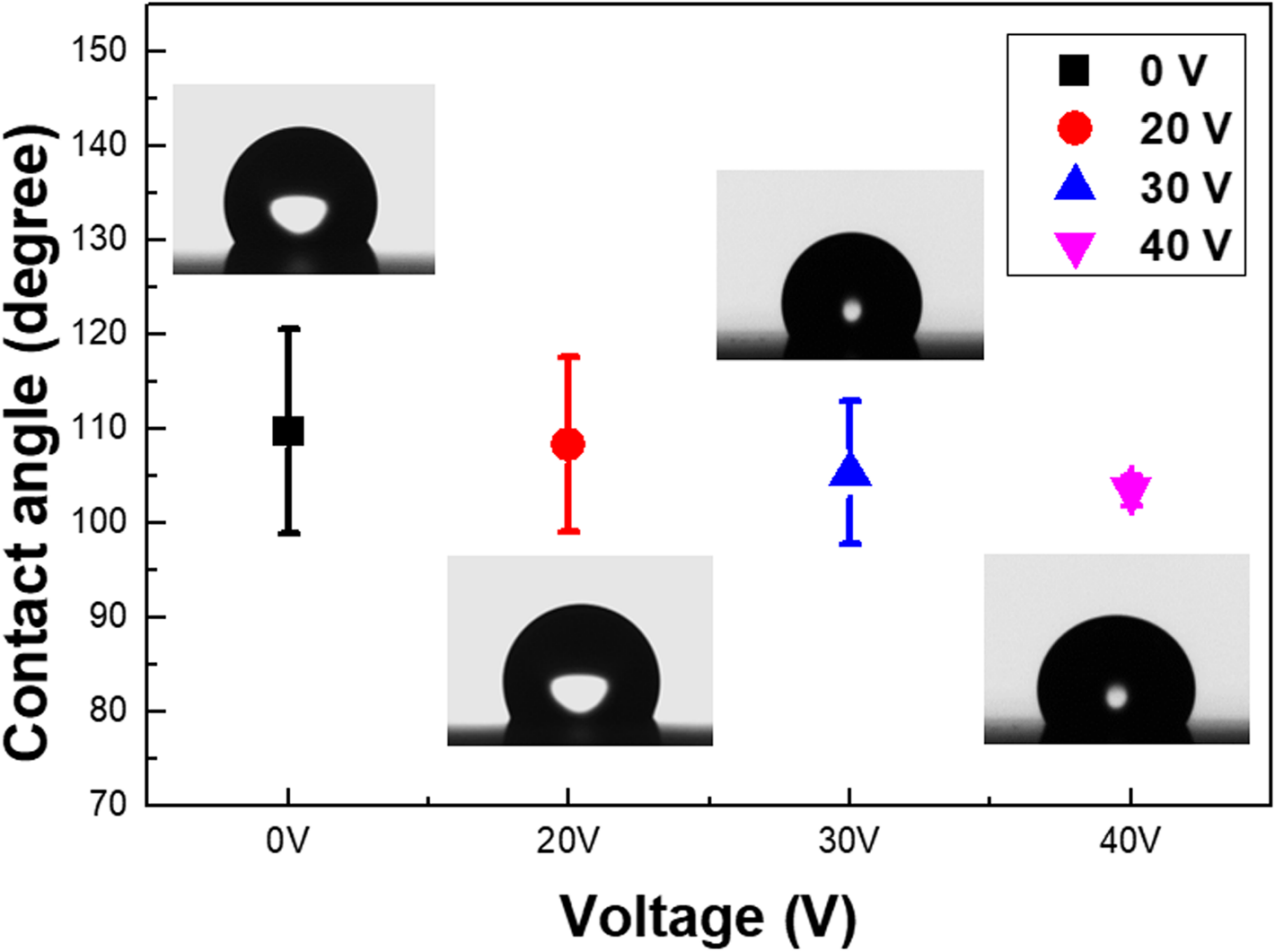
Contact-angle measured results of Si surfaces

In addition, the surface wettability of prepared Si textures was examined, where all the samples were measured by six times at different positions, as presented in Fig. 4. It has been reported that the measured contact angle was correlated with the roughness of nanostructures according to the equation shown below [24, 25],

2 $$ \mathrm{cos}\uptheta ={\mathrm{Rfcos}\uptheta}_{\mathrm{e}}-\mathrm{R}\left(1-\mathrm{f}\right) $$

in which *θ* and *θ*_e_ are the contact angle of rough and flat Si surfaces, respectively, and *R* represents the roughness factor. In addition, *f* is the area fraction of the air/water surfaces. It could be found that the average contact angles from four different samples were 109.8^0^ ± 10.8^0^ in the case of etching without bias, 108.4^0^ ± 9.2^0^ with + 20 V of bias, 105.4^0^ ± 7.6^0^ with + 30 V of bias and 103.6^0^ ± 1.6^0^ with + 40 V of bias, as shown in Fig. 4. The greatly reduced deviation at measured contact angle from the employment of + 40 V could be attributed to the relatively uniform topography on etched surfaces, which indicated that the utilization of bias in MaCE not only enabled to enhance the etching rate, but could further sustain the sound etching uniformity that was essential for the practical applications.

**Fig. 5:**
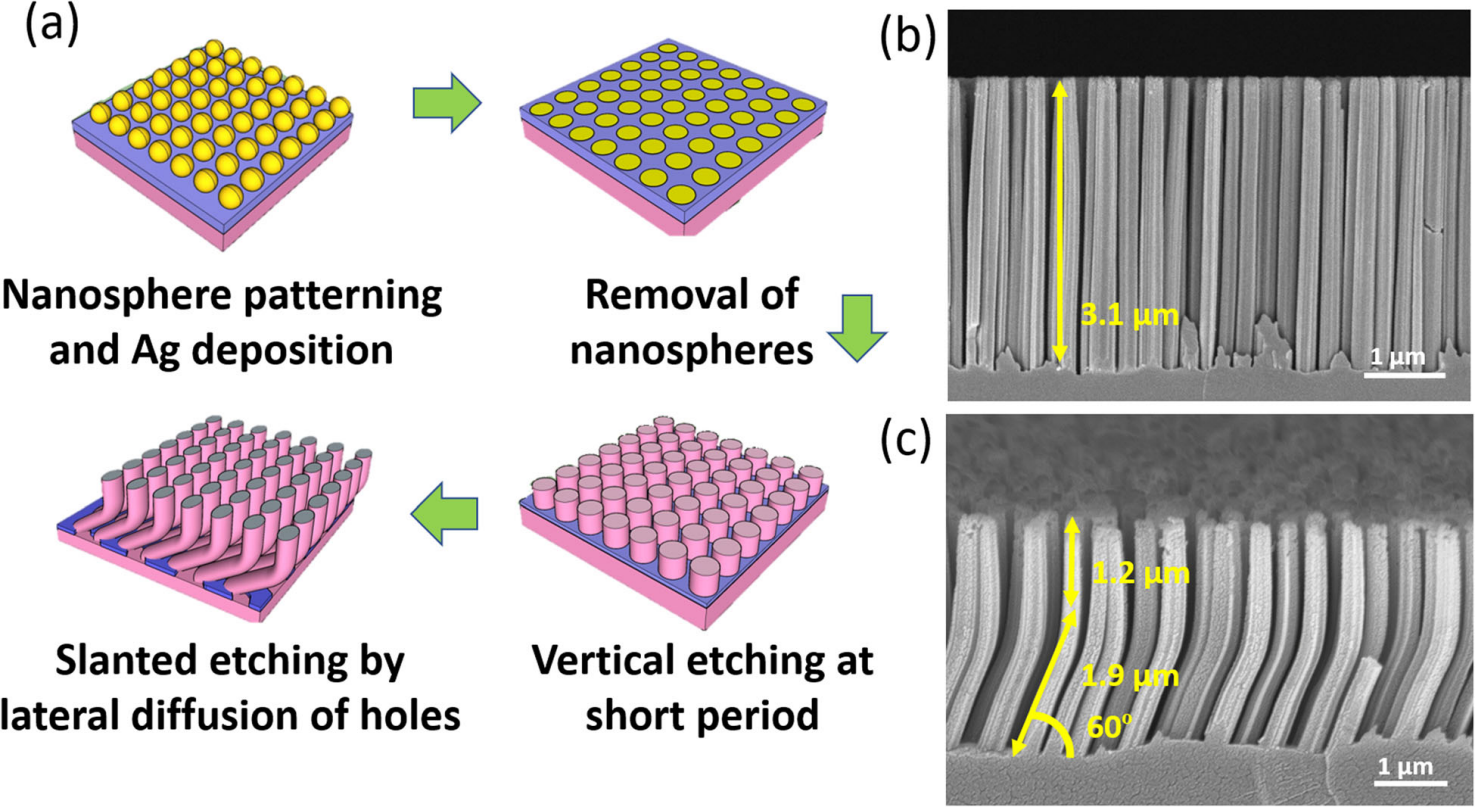
**a** Schematic illustration of process flow for the formation of slanted/vertical SiNW arrays. Cross-sectional SEM images of **b** vertical SiNWs and **c** slanted/vertical SiNWs

In addition to the modulation of etching rates, the etching orientation could be also manipulated with the external bias, as shown in Fig. 5a. In this test, the combination of MaCE with nanosphere lithography was conducted for defining the Ag patterns through self-assembled polystyrene nanospheres [26]. By applying the vertical bias relative to the substrate planes during MaCE process, the directional etching along the bias orientation was created, where the vertically regulated SiNW arrays were formed, as presented in Fig. 5b. Switching the etching direction from the vertical feature toward a slanted profile was realized by varying the electric polarization at 60^°^ with respect to the in-plane direction. While the bias was modulated, it overall resulted in the formation of two-segment slanted/vertical SiNW arrays, as presented in Fig. 5c.

**Fig. 6:**
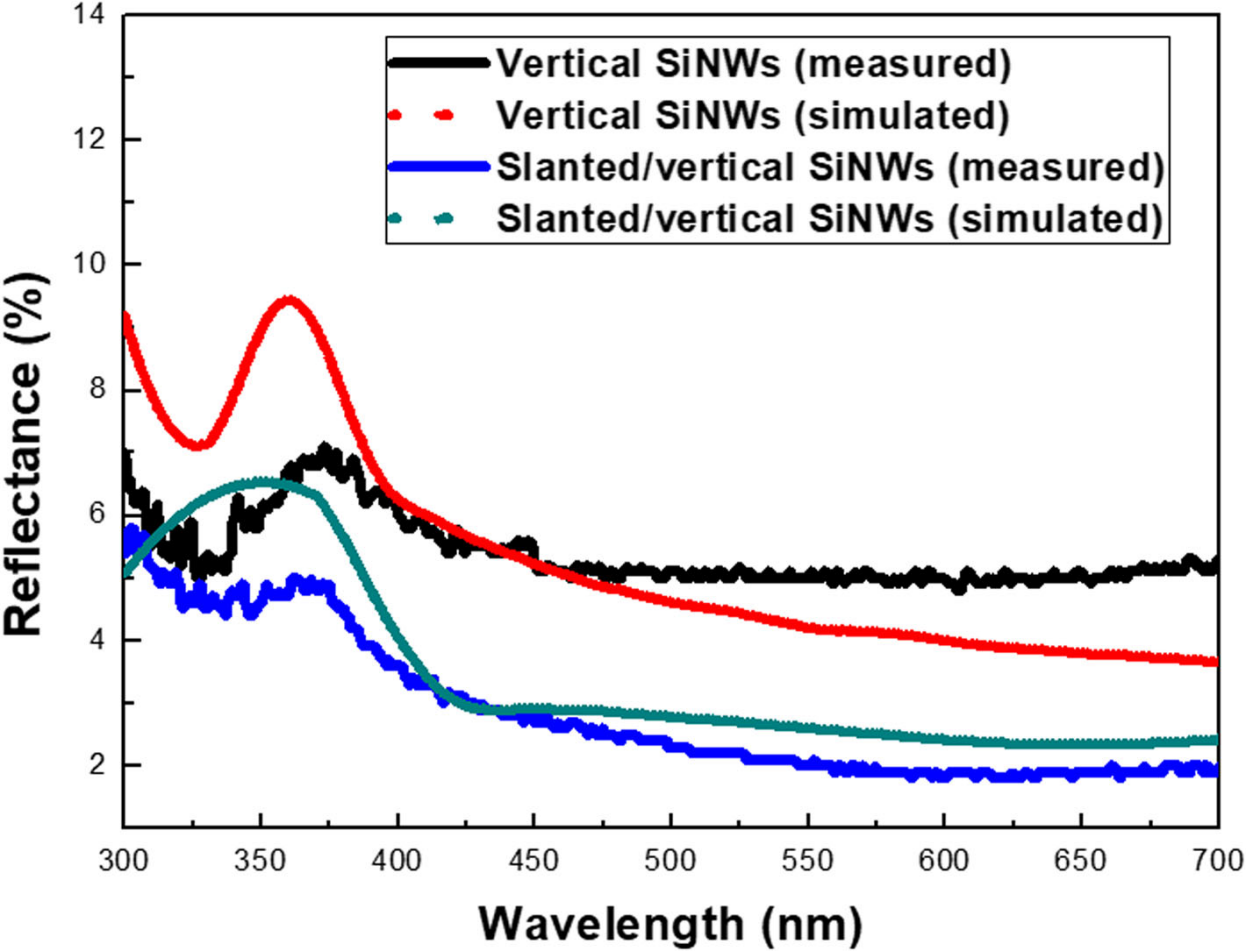
Measured and simulated reflection results of both vertical and slanted/vertical SiNW arrays, respectively

Unlike the typical way to vary the NW orientation through the diffusion-controlled local change of etchant concentrations in the etching condition [27, 28], here both the concentrations of oxidants and etchants remained consistent and thus, the variation of etching direction was mainly contributed from the external bias. This might give rise to the feasibility for the realization of three-dimensional processing capability. Finally, the visible reflectance was examined from both vertical and two-segment based SiNW arrays, as demonstrated in Fig. 6. The measured reflection results clearly verified that the slanted/vertical SiNW arrays with average reflectance of 2.8% possessed the comparably lower light-reflection capability than that of sole vertical SiNW arrays (average reflectance = 5.4%) covering the visible bands. To further confirm the experimental investigation, the simulated reflection results were also compared, as shown in Fig. 6. It could be found that the simulated findings corresponded well to the measured results, indicating the superior antireflection characteristics of slanted/vertical nanostructures.

## Conclusion

Electric-field assisted MaCE method for the formation of orientation-controlled SiNW arrays with improved etching rate was presented. The underlying mechanism was elucidated by the combined effects of hole rectification and anodization that could modulate the etching morphologies and kinetics. Furthermore, the surface wettability was examined, indicating that the large-area uniformity was created while the bias was + 40 V. By manipulating the polarization of applied electric field, the transition of the etching direction from the vertical feature toward slanted profile was realized. Such two-segment SiNWs in the form of slanted/vertical features possessed the greatly improved antireflection properties, which might be potentially useful for optoelectronic devices, photonic crystals, and other multifunctional applications.

## Additional File

**Additional file 1: Figure S1.** Schematic illustrations for conducting bias-assisted MaCE process. **Figure S2.** Cross-sectional SEM images of SiNW arrays made by various bias-assisted MaCE process. The Ref represented the case without applying bias.

## Data Availability

The datasets supporting the conclusions of this article are included within the article.

## Abbreviations

FDTD: Finite-difference time-domain
MaCE: Metal-assisted chemical etching
NSL: Nanosphere lithography
PL: Photoluminescent
PSs: Polystyrene nanospheres
SEM: Scanning electron microscope
SiNWs: Silicon nanowires
