# Supplementary material for: High-Speed and Direction-Controlled Formation of Silicon Nanowire Arrays Assisted by Electric Field
Source: Nanoscale Res Lett. 2020 Jan 30;15:25. doi: 10.1186/s11671-020-3259-5 (PMC6990330; doi:10.1186/s11671-020-3259-5)
Supplement: Supplementary file 1 — Additional file 1: Figure S1. Schematic illustrations for conducting bias-assisted MaCE process. Figure S2. Cross-sectional SEM images of SiNW arrays made by various bias-assisted MaCE process. The Ref represented the case without applying bias. [file 11671_2020_3259_MOESM1_ESM.docx]

**Additional files**

**High speed and direction-controlled formation of silicon nanowire arrays assisted by electric field**

Pin-Ju Chien^1^, Ta-Cheng Wei^1^, Chia-Yun Chen^12*^

^1^Department of Materials Science and Engineering, National Cheng-Kung University, Tainan 701, Taiwan

^2^Hierarchical Green-Energy Materials (Hi-GEM) Research Center, National Cheng Kung University, No.1 University Road, Tainan 701, Taiwan


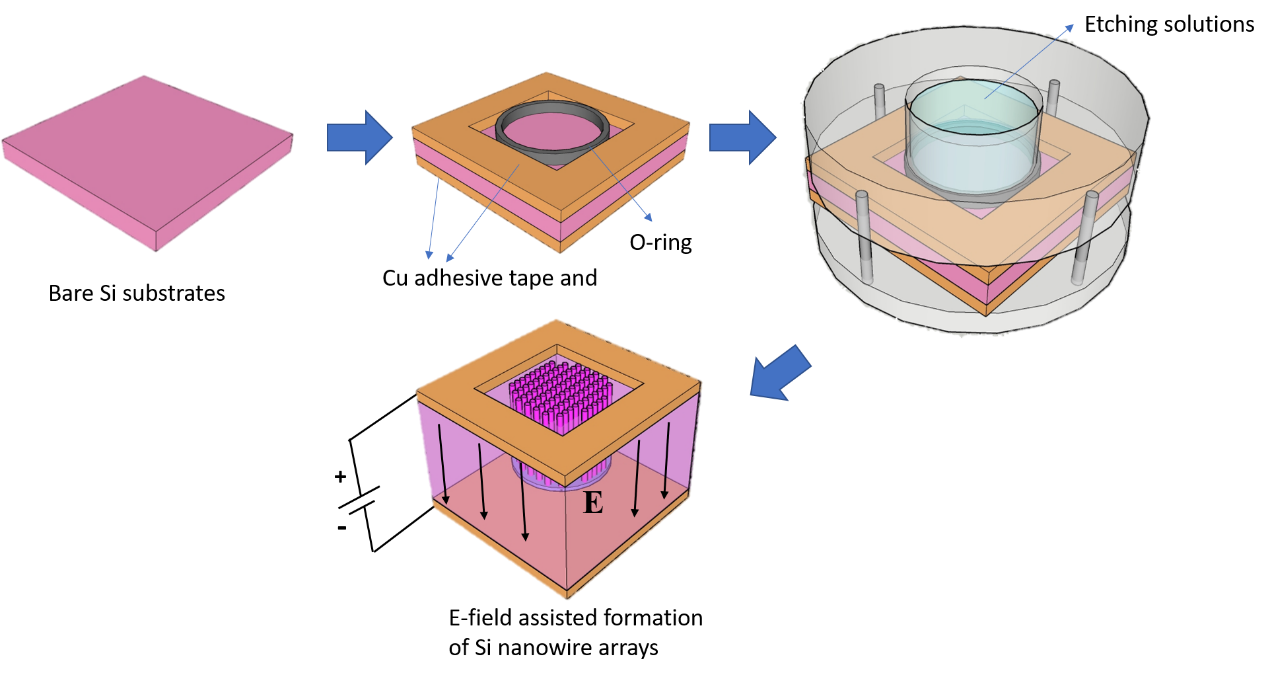


Fig. S1 Schematic illustrations for conducting bias-assisted MaCE process.


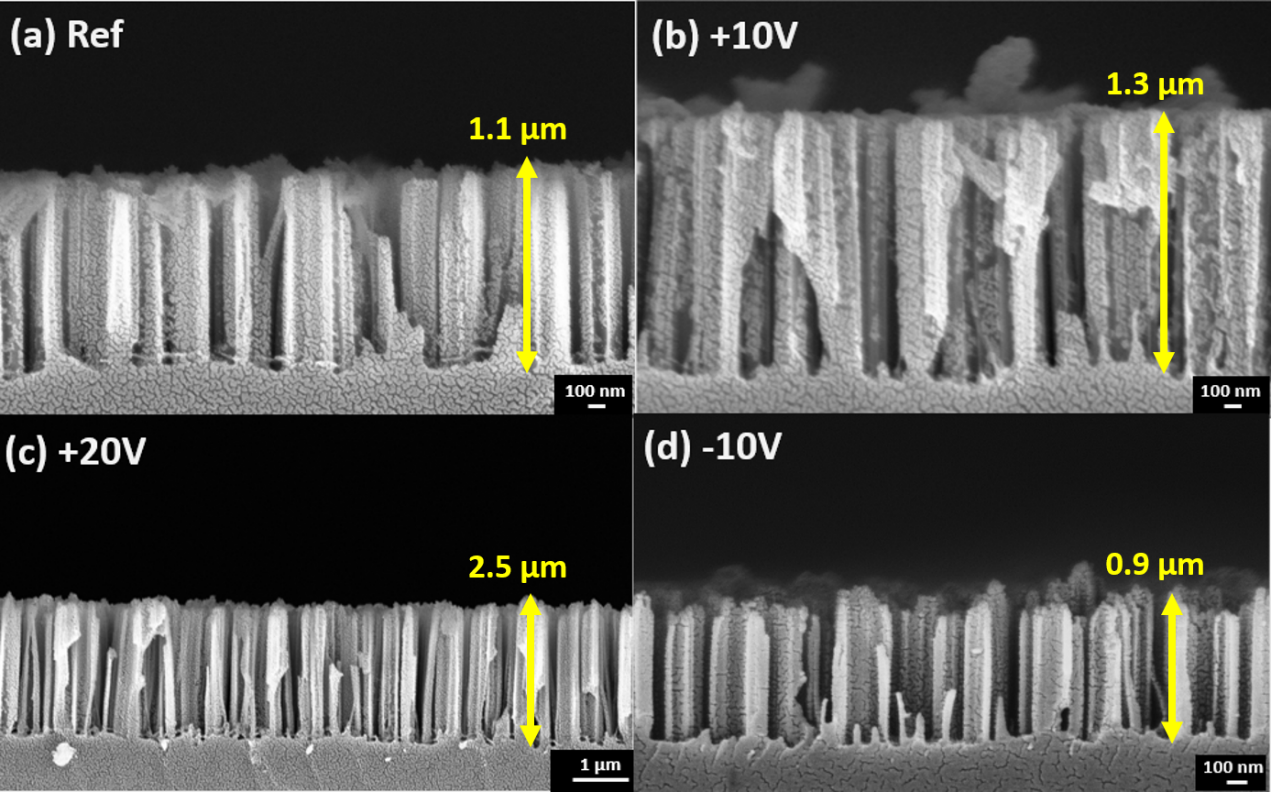


Fig. S2 Cross-sectional SEM images of SiNW arrays made by various bias-assisted MaCE process. The Ref represented the case without applying bias.
